# Testing Local Host Adaptation and Phenotypic Plasticity in a Herbivore When Alternative Related Host Plants Occur Sympatrically

**DOI:** 10.1371/journal.pone.0079070

**Published:** 2013-11-12

**Authors:** Lorena Ruiz-Montoya, Juan Núñez-Farfán

**Affiliations:** 1 Grupo Ecología Evolutiva y Conservación, Departamento de Conservación de la Biodiversidad, El Colegio de la Frontera Sur, San Cristóbal de Las Casas, Chiapas, México; 2 Laboratorio de Genética Ecológica y Evolución, Departamento de Ecología Evolutiva, Instituto de Ecología, Universidad Nacional Autónoma de México, Distrito Federal, México; University of Massachusetts, United States of America

## Abstract

Host race formation in phytophagous insects can be an early stage of adaptive speciation. However, the evolution of phenotypic plasticity in host use is another possible outcome. Using a reciprocal transplant experiment we tested the hypothesis of local adaptation in the aphid *Brevicoryne brassicae*. Aphid genotypes derived from two sympatric host plants, *Brassica oleracea* and *B. campestris*, were assessed in order to measure the extent of phenotypic plasticity in morphological and life history traits in relation to the host plants. We obtained an index of phenotypic plasticity for each genotype. Morphological variation of aphids was summarized by principal components analysis. Significant effects of recipient host on morphological variation and life history traits (establishment, age at first reproduction, number of nymphs, and intrinsic growth rate) were detected. We did not detected genotype × host plant interaction; in general the genotypes developed better on *B. campestris*, independent of the host plant species from which they were collected. Therefore, there was no evidence to suggest local adaptation. Regarding plasticity, significant differences among genotypes in the index of plasticity were detected. Furthermore, significant selection on PC1 (general aphid body size) on *B. campestris*, and on PC1 and PC2 (body length relative to body size) on *B. oleracea* was detected. The elevation of the reaction norm of PC1 and the slope of the reaction norm for PC2 (i.e., plasticity) were under directional selection. Thus, host plant species constitute distinct selective environments for *B. brassicae*. Aphid genotypes expressed different phenotypes in response to the host plant with low or nil fitness costs. Phenotypic plasticity and gene flow limits natural selection for host specialization promoting the maintenance of genetic variation in host exploitation.

## Introduction

Host race formation or local host adaptation in phytophagous insects is considered an early stage of adaptive speciation in sympatry [1]–[6]. However, local adaptation to a host plant might be limited by the herbivore’s genetic diversity, gene flow and/or phenotypic plasticity [3], [7]. Absence of genetic variation for host use (i.e., traits related to it) impedes selection to fit the best phenotype in each host. In turn, phenotypic plasticity implies that alternative phenotypes are produced by genotypes due to their environmental sensitivity [8], [9].

Phytophagous insects commonly interact with different host species at a local scale. Even at this scale, local adaptation to different host plants species is an expected outcome if selection in each host species is strong enough to prevent the homogenizing effects of gene flow. However, under soft selection (the probability of breeding after migration, cf. [10]), the plastic genotypes could be favoured over the local specialist genotypes [11]–[13]. Genotypes may respond differentially to each host by producing different phenotypes, those with the most favourable phenotype in each host persisting while others go extinct [14], [15]. Although a single genotype might produce the best phenotype in all available hosts, plasticity may also induce a deviation far from the best local phenotype, reducing relative fitness of plastic genotype. Therefore, even though plasticity can potentially offer the best solution to herbivores in terms of phenotypic optima in different environments, costs and limits of plasticity may render plasticity suboptimal [16] or even maladaptive. The benefits of plasticity arise from phenotype-environment matching across different environments. Thus, adaptive genetic differentiation and adaptive phenotypic plasticity are two evolutionary paths to maximize fitness in response to environmental heterogeneity, and these responses are not mutually exclusive [17], [18].

To test the importance of phenotypic plasticity versus local adaptation in the interaction between a herbivorous insect and different host plants we studied the cabbage aphid, *Brevicoryne brassicae* Linnaeus (Homoptera: Aphididae), which feeds on host plants of Brassicacea family [19]. In the state of Chiapas, south-eastern Mexico, this aphid reproduces parthenogenetically [20]. Two common host plants of *B. brassicae* in this region are *Brassica campestris* L. and *Brassica oleracea* L., the former a superior host in terms of aphid fecundity and size [21], [22]. Although differences in the probability of successful of establishment have been detected for aphids derived from different field hosts, suggesting local adaptation [23]; selection on the aphids reproductive span in each host is similar for aphids from both origins [20]. Overall, the genetics of the aphid populations in the highlands of Chiapas revealed a moderate genetic structure (*F*_st_ = 0.22), however, very little genetic structure was explained by the host plant (*F*_st_ = 0.03) [24]. Therefore, in this study, we tested the hypothesis of local adaptation in *B. brassicae* and assessed how the magnitude of phenotypic plasticity in morphological and life history traits is affected by selection. We analysed morphological and life history traits of clones (i.e., genotypes) derived from 28 aphid females collected and reared on both *Brassica campestris* and *B. oleracea* hosts.

## Materials and Methods

### Biological System of Study

*Brevicoryne brassicae*, an aphid species of palaearctic origin, is widely distributed and closely associated with host plant species of the Brassicaceae family [19]. This aphid reproduces both sexually and asexually in cold regions of the world and completes its life cycle without host alternation. In regions with mild winters, like Chiapas, Mexico, reproduction is only by parthenogenesis [19].

*B. brassicae* and its cultivated host species *B. oleracea* (green cabbage) were probably simultaneously introduced to Mexico *ca*. 100 years ago [25]. In the Chiapas highlands, the weed *B. campestris* and the cultivated *B. oleracea* var. *capitata* (green cabbage) are the main host plants of *B. brassicae* and both plant species commonly occur in sympatry. Green cabbage is cultivated from the end of autumn (November) to the end of winter (February). *B. campestris* is an annual weed that grows nearby from cultivated fields of *B. oleracea*. After harvest, *B. campestris* is tolerated and hence invades the fallow fields. In summer, small populations of *B. brassicae* can be found on discarded plants of *B. oleracea* during harvest or on late-emerging *B. campestris* plants. These host species present different environments for *B. brassicae* as *B. campestris* has a higher content of glucosinolates and higher leaf and stem trichome density than *B. oleracea* [25]. In addition, *B. campestris* has a higher content of leaf nitrogen than *B. oleracea* [22].

### Rearing Conditions

Plants of *B. campestris* were grown from seeds collected in a field in the town of Teopisca (92°28′25″ W; 16°38′19″ N; 1800 m; annual average temperature, 17.25°C; annual average precipitation, 700 mm) (permission granted by Manuel Girón Intzín). *B. oleracea* seeds were obtained from a local farmer. Plants of both species were individually grown in pots in the greenhouse. Three seeds were sown in pots (20 d×30 h cm) with sterilized soil, and randomly assigned to benches in the greenhouse. One plant per pot was used to produce aphid clones or for performance tests. Each plant was covered with a mesh to exclude aphids and natural enemies [19]. A total of 240 plants per species were produced. Plants were kept at ambient temperature and photoperiod (13±4°C and 12 h daylight), without fertilization, and watered every other day.

### Life History Characters

A single parthenogenetic *B. brassicae* female was collected from each of twenty *B. campestris* plants and *B. oleracea* plants in Teopisca, making a total of 40 females. Each female and their offspring produced after one month were considered a genotype. From each genotype (clone) six nymphs were individually placed on *B. campestris* plants and six on the same number of *B. oleracea* plants. Each plant was covered with mesh. Each nymph was observed daily to determine if it successfully established itself on a host plant (i.e., ability to reproduce), as well as record its age at first reproduction, and the number of nymphs laid during a 15 days period of reproductive life (reproduction) (Table S1). A previous study demonstrates that most reproduction occurs during the 13 days period subsequent to onset of reproduction [23]. Morphological and life history traits were not obtained for unsuccessful females and sample size was reduced for these traits. Seven genotypes collected on *B. campestris* (7/20) and five from *B. oleracea* (5/20) failed to establish successfully on one or both hosts.

We calculated the intrinsic growth rate per individual aphid according to the formula *r*_m_ = 0.738 [(ln*M*_d_)/*d*], where *d* is age at first reproduction and *M*_d_ is the number of descendants during a period of 15 days [26].

### Morphological Traits

At the end of the experiment, each female was collected and kept in alcohol (70% solution). Then it was placed in a 10% KOH solution for 40 min, rinsed every half-hour during 8 h with distilled water, and cleared in a solution of chloral hydrate-phenol (1∶1) for at least 24 h ([19]. Finally, specimens were placed on a slide; a drop of Berlesse medium was added and a cover glass was applied. Slides were oven-dried at 40°C for two weeks. Aphids were measured using a stereomicroscope (Stemi V6, Carl Zeiss). The length of body (BL), antennal segment III (ASIII), and hind tibia (HT) segment were measured (Table S1). These characters were chosen because they are involved in the recognition and use of the host plant [27], or associated host plant quality [21], [23]. Variation in morphology was summarized by principal components analysis (PC).

### Data Analysis

#### Genetic differentiation between host species

Differences among genotypes in relation to the host plant species were assessed using a mixed model analysis of variance (Anova) of age at first reproduction, number of nymphs, intrinsic growth rate, and on the scores of two principal components (PC1 and PC2). The variables age at first reproduction and number of nymphs were *ln* transformed to approximate experimental error to a normal distribution. The source host (i.e., origin) and recipient host (i.e., where grown) were declared as fixed effects, while genotype as a random effect nested within the source host. The host plant was declared as a fixed factor because the study was aimed to test local adaptation to these two particular host species known to elicit aphid variability in morphological and life history characteristics and occur in the same cultivated fields. Interactions between explanatory variables were included [28], [29]. Establishment was analysed using a nominal logistic fit model. The response variable was recorded as successful (1) or unsuccessful (0) establishment as a function of the same independent variables described above. Significance of the effects was tested by Likelihood-ratio Chi-square tests [30]. All analyses were carried out using the statistical software JMP 5.1.2. [31].

The interaction term source × recipient host in both the Anovas and the nominal logistic regression was of primary interest because it characterises genetic variation in the responses to recipient host. Differences among genotypes indicate genetic variation for the traits. Differences between recipient hosts suggest that these are different environments that affect performance of *B. brassicae*. The interaction genotype (nested in source host) × recipient host can be interpreted as evidence for local adaptation [15], if the effects are in the expected direction.

#### Phenotypic plasticity

Phenotypic plasticity in morphological and life history traits of *B. brassicae* was analyzed by obtaining an index of plasticity for each genotype following Valladares et al. [32], calculating all pair-wise differences between individuals within a clone between environments (i.e., the two hosts). The index of plasticity was obtained for each character, for each genotype. This index is particularly relevant in this system because each aphid was allocated to one individual plant, representing a potentially additional environmental source of phenotypic variation within genotypes. The differences represent phenotypic plasticity for a given trait among individuals with the same genotype but that developed on different plants. A nested Anova of the Valladeres’ index of plasticity of each morphological (PC1 and PC2) and life history trait (age at first reproduction, number of nymphs laid, and intrinsic growth rate) in relation to the host of origin, and genotype nested within the host of origin, was performed to determine potential differences in plasticity. A significant host of origin effect indicates differences in the average index of plasticity between aphids collected on each plant, and significant genotype effect indicates differences in plasticity among genotypes.

#### Natural selection on morphological plasticity

The reaction norm of each genotype for PC1 and PC2 was characterized by its elevation and slope [16], [33], [34]. We regressed the average values of PC1 and PC2 of each genotype against the environments (host plants), declaring host as a continuous variable to obtain the slope and elevation of the reaction norm for each morphological trait per genotype. Slope and elevation represent the magnitude of phenotypic change induced by the hosts and the average trait value, respectively [34].

We tested if the slope and elevation of the reaction norm is related to the average relative fitness of genotypes by means of a multiple linear regression model [13], [16], [35]. Relative intrinsic growth rate of each aphid was our estimator of fitness. Under this framework, a positive relationship between fitness and elevation is interpreted as the presence of selection for the maximum average body size across environments, whereas a positive relationship between the slope and fitness, suggests selection on body size plasticity; a negative relationship implies constraint plasticity [16]. Regression analysis of the reaction norm components in relation to fitness has the potential problem of co-linearity [36]. We explored these relationships by correlation analysis of trait plasticity and average trait values of genotypes within hosts [36], as well as randomization analysis of selection gradients as suggested by Roff [37].

The presence of plasticity costs was assessed by regression analysis of residual fitness (after regressing fitness against trait mean value) as a function of plasticity of the genotypes; a negative correlation would be indicative of costs [16]. Fitness was the mean intrinsic growth rate across hosts, and the slope of the reaction norm was the magnitude of plasticity. This analysis was conducted for PC1 and PC2 separately.

In order to determine if phenotypic plasticity results from selection acting independently in each host, we estimated the selection gradient on PC1 and PC2 in each host plant species [38]. We estimated the selection gradient by multiple regression analysis using standardized scores of PC1 and PC2 for each individual. Individual’s relative fitness was estimated as the intrinsic growth rate divided by the population mean intrinsic growth rate, thus 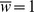. All analyses were performed using the JMP 5.1.2. statistical software [31].

## Results

### Morphological and Life History Variation

**Figure 1:**
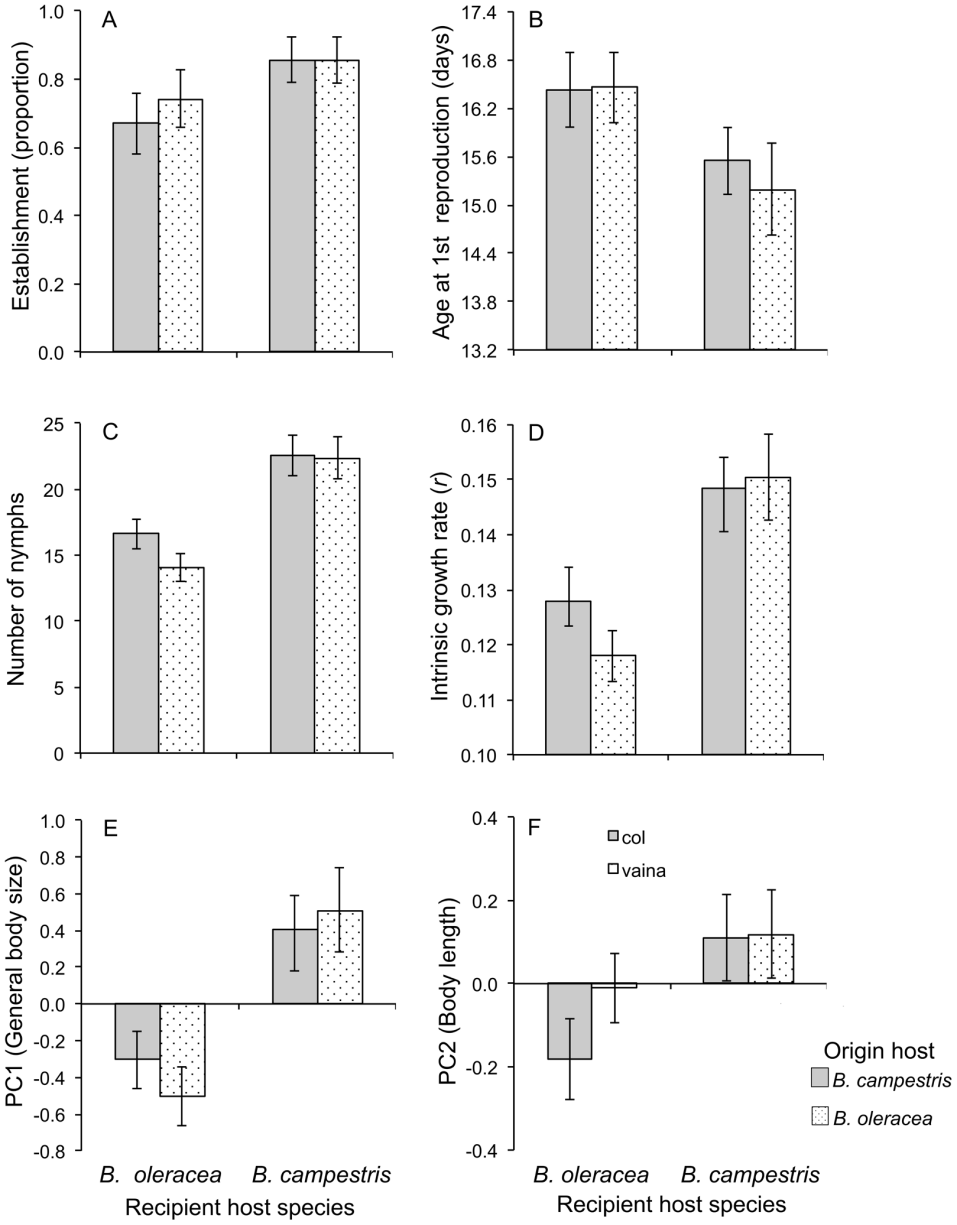
Average values (± SE) of morphological (PC1 and PC2) and life history traits of populations of *Brevicoryne brassicae* associated with *Brassica oleracea* and *Brassica campestris*.

Aphids’ morphological variation was summarized by PC1 and PC2, accounting 86.5% of the variance. PC1 accounted for 67% of variation (general body size), while 18.9% of variation (shape, size of appendages in relation to body size) was attributable to PC2 (Table 1). The general body size (PC1) and body length (PC2) had greater values in *B. campestris* than in *B. oleracea* (Figure 1 A, B).

**Table 1:** Principal components analysis of three morphometric characters of females of *Brevicoryne brassicae* reared on *Brassica campestris* and *Brassica oleracea* (N = 233).

Trait	PC1	PC2
Body length	0.55	0.81
Length antennae segment III	0.58	−0.55
Hind tibia	0.60	−0.21
Eigenvalue	2.03	0.57
Percent of variance	67.51	19.00
Cumulative percent	67.5	86.5

Successful establishment of aphids was *ca*. 60% in *B. oleracea* and 80% in *B. campestris*, independent of host of origin (Fig. 1C). Genotypes differ in their probability to establish successfully (nested within origin; Likelihood ratio test, χ^2^ = 63.39, *d.f*. = 35, *P* = 0.0023) and interacted with the recipient host (Likelihood ratio test χ^2^ = 53.266, *d.f.* = 35, *P* = 0.024).

**Figure 2:**
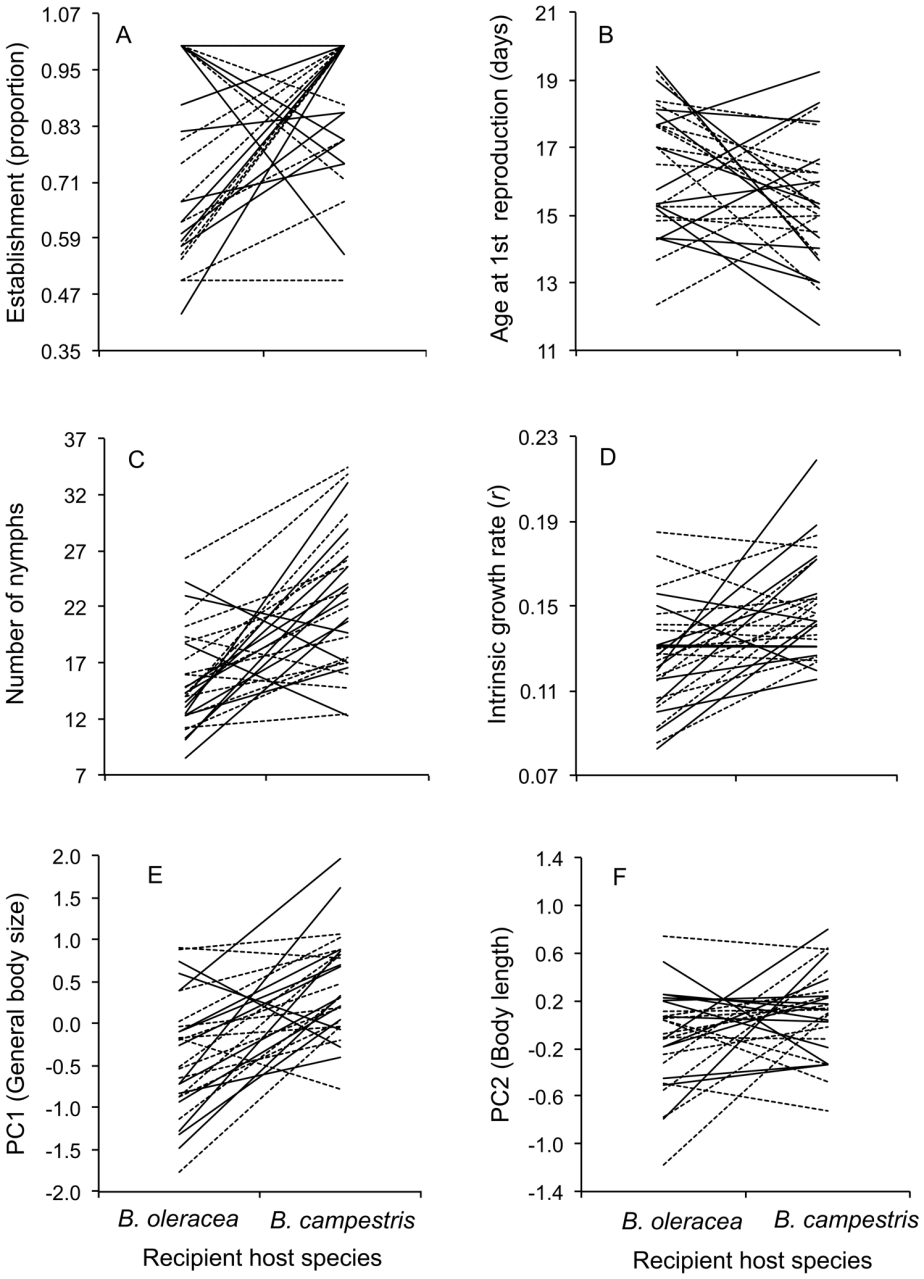
Reaction norms of 27 genotypes of *Brevicoryne brassicae* reared in *Brassica campestris* and *B. oleracea*. Points are the average value of 4–5 individuals of the same genotype. Genotypes were collected from *B. campestris* (solid lines) and *B. oleracea* (dashed lines).

A significant recipient host effect was detected for all life history traits (Table 2). On average *B. brassicae* females initiated reproduction 1.5 days earlier and laid more nymphs on *B. campestris* than on *B. oleracea* (Fig. 1B, D). Similarly, the number of nymphs and intrinsic growth were higher in *B. campestris* than in *B. oleracea* (Fig. 1 E, F). In all cases, the effect of the origin host, genotype, and interactions (Fig. 2) were not significant (Table 2).

**Table 2:** Statistic *F* from analysis of variance of morphological (PC1, PC2) and life history traits of *Brevicoryne brassicae* reared on hosts *Brassica campestris* and *Brassica oleracea*.

Source of variation	*d.f.*	Age at first reproduction	Number of nymphs	Intrinsic growth rate	PC1	PC2
Origin host (OH)	1	0.073 ns	0.786 ns	0.217 ns	0.004 ns	0.725 ns
Recipient host (RH)	1	4.543*	20.307**	16.072**	27.594**	4.001 =
Genotype nested in OH (G(OH))	26	1.432 ns	1.267 ns	1.521	1.619 ns	1.038 ns
OH×RH	1	1.432 ns	0.362 ns	0.988 ns	0.874 ns	0.332 ns
G (OH)×RH	26	0.969 ns	1.063 ns	1.018 ns	0.72 ns	1.074 ns

*d.f.* = Degrees of freedom;

* *P*<0.05;

** *P*<0.001;

= P = 0.05;

ns, not significant.

### Phenotypic Plasticity of Morphological and Life History Traits

The origin host did not affect the phenotypic plasticity index of *B. brassicae* genotypes (Table 3). In contrast, significant differences between aphid genotypes regarding the index of plasticity for all characters were detected (Table 3).

**Table 3:** Statistics *F* from analysis of variance of plasticity index of life history traits of 27 genotypes of *Brevicoryne brassicae* collected and grown on *B. campestris* and *B. oleracea*.

Source variation	*d.f.*	Age at first reproduction	Number of nymphs	Intrinsic growth rate	PC1	PC2
Genotype	26	3.364*	2.738*	3.684*	3.393*	3.053*
Origin host	1	0.186 ns	2.522 ns	0.738 ns	1.036 ns	0.756 ns

* *P*<0.05; ns, not significant.

### Selection of Morphological Traits

In each recipient, host positive directional selection on PC1 was detected (β*_B. campestris_* = 0.087; (β*_B. oleracea_* = 0.138). Aphids with larger body size (PC1) achieve more fitness; an increase in body size (PC1) by one standard deviation is expected to result in 8.7% more fitness than mean individual fitness in *B. campestris* (*R* ^2^ = 0.055) and 13.8% in *B. oleraceae* (*R* ^2^ = 0.152) (Table 4). Selection on PC2 on both recipient hosts was not significant, however it was marginally significant on *B. oleracea* (Table 4). Average mean relative fitness was higher on *B. campestris* (Fig. 1F).

**Table 4:** Lineal regression analysis of relative fitness as a function of morphological traits (PC1 and PC2) of *Brevicoryne brassicae* from two host plants.

Host	Trait	Directional selection gradients, *ß.*	Anova of regression model				
			SV	*d.f*	MS	*F*	R^2^
*Brassica campestris*	PC1	0.087 (0.03)**	Model	2	0.42	4.16*	0.055
	PC2	0.014 (0.03) ns	Error	106	0.101		
							
*Brassica oleracea*	PC1	0.138 (0.029)***	Model	2	1.266	12.06***	0.152
	PC2	0.052 (0.029) ns	Error	121	0.104		

Standard error of *ß* is given in paréntesis.

SV = Source of variation;

* *P*<0.05;

** *P*<0.01;

*** P<0.002;

ns, not significant.

### Selection on Phenotypic Plasticity

The regression analysis of relative fitness on the reaction norm of PC1 was significant for the elevation (mean trait value; *P* = 0.052) but not for its slope (trait plasticity; *P* = 0.22). In contrast, positive directional selection on the slope of the reaction norm of PC2 was marginally significant but not for its elevation (Table 5). The bootstrap analyses show a potential distribution of selection gradients on plasticity from −0.029 to 0.181 for PC1, and from 0.028 up to 0.195 for PC2 (plasticity) (Table 5). The correlation of fitness residuals with the degree of plasticity was not significant for PC1 (R^2^ = 0.02, *ß* = 0.003, *P* = 0.48) or PC2 (R^2^ = 0.083, *ß* = 0.009, *P* = 0.14).

**Table 5:** Selection gradients (SE) on the components of the reaction norm (slope and elevation) of morphological traits of *Brevicoryne brassicae* in response to two host plants.

Reaction norm component	*ß*	Confidence interval of *ß* (at 95%)	
		Minimum	Maximum
PC1 elevation	0.096 (0.047) =	−0.012	0.181
PC1 slope	0.039 (0.031) ns	−0.029	0.114
PC2 elevation	0.042 (0.088) ns	−0.172	0.191
PC2 slope	0.093 (0.046) =	0.028	0.195

Confidence interval of *ß* was obtained through bootstrapping. = , *P* = 0.05.

**Figure 3:**
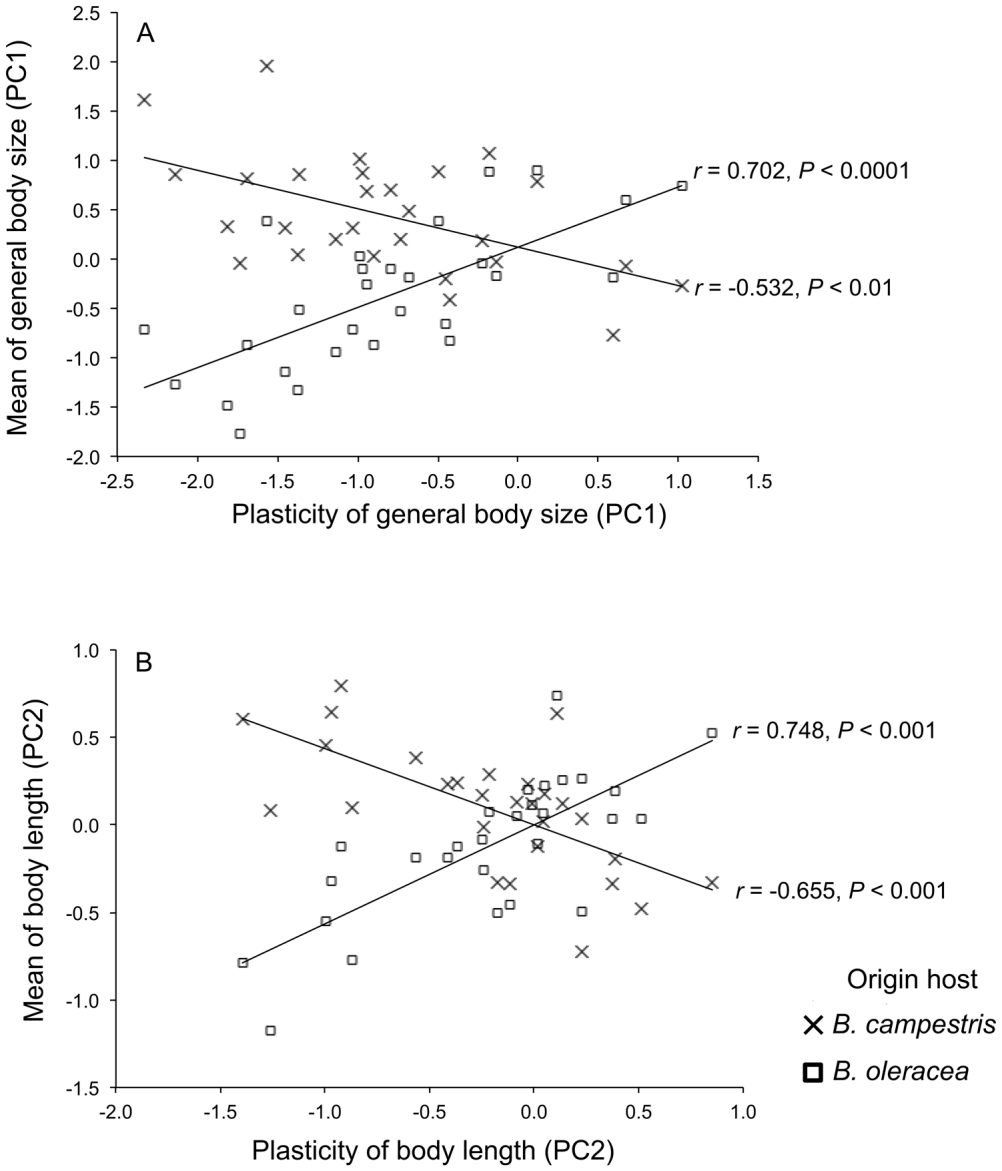
Relationship between trait value and plasticity of two morphological traits of *Brevicoryne brassicae*. (A) PC1, general body size; (B) PC2, body length. The points represent the average value per genotype.

Correlation analysis of average trait value (mean trait) and trait plasticity (slope of reaction norm) differed between recipient hosts. This correlation was positive for PC1 and PC2 in *B. oleracea* but negative in *B. campestris* (Fig. 3).

## Discussion

For the most part, aphids grown on *B. campestris* attained, in general, larger body sizes (PC1) and relative body length (PC2) than those grown on *B. oleracea,* independent of genotype or host plant of origin. Both *B. brassicae* host plant species constitute distinct selective environments for *B. brassicae*, however the plastic response seems to involve low or nil fitness costs and plasticity in PC2 is positively selected (see [18]). Phenotypic plasticity and gene flow limits natural selection for host specialization promoting the maintenance of genetic variation in host use.

In the Chiapas highlands populations of *B. brassicae* are not locally adapted to host plants species, *B*. *campestris* and *B. oleraceae*, because local herbivore populations did not outperform the foreign ones. Aphids of both origins performed better when grown on *B. campestris*. Aphids morphological variation are determined by the particular host plant (*B. oleracea* and *B. campestris*) either in the field [21], or under experimental greenhouse conditions [39]. In general, aphids grown on *B. campestris* are relatively thicker with long appendages, while aphids grown on *B. oleracea* are slightly flattened with short appendages. These phenotypes are elicited and predictable in these two *Brassica* species independent of the aphids’ genotype or their host plant of provenance. The difference in nitrogen content found between *B. oleracea* and *B. campestris* has been implicated as a source of phenotypic variation in *B. brassicae* [21]. However a greenhouse experiment, in which the host plants were supplemented with soil nitrogen, failed to detect differences between hosts with regard to the elicited aphids’ morphological phenotype, but positively affected the number of nymphs laid [39]. Plants’ phosphorous, potassium and glucosinolate content, which varies between hosts [40]–[42] has been shown to affect aphids’ development [43]–[45]. Thus it would be important to test if these play a role in the plastic morphology of *B. brassicae*.

Aphid genotypes of *B. brassicae* differed in their plasticity, but the Valladares’ index of plasticity was similar for aphids derived from *B. campestris* or *B. oleracea*. Since each individual female was established on a single plant, the plasticity index of a given genotype encompasses a fraction of the phenotypic variation elicited within a host plant species. Therefore, *B. brassicae* is not only highly sensitive to inter-specific but also to intra-specific differences among host plants. Can the distribution of phenotypic plasticity be partitioned in intra- and inter-specific components? Finding an answer to this question requires experimentally controlling the genetic variation of hosts, such that each aphid genotype develops on plants with known genotypes or the use of lines selected for low and high glucosinolates content [46]. Thus, within-host variation precludes the detection of a genotype×host interaction.

Local adaptation to a host plant can be inferred from G×E interaction, with genotypes performing better in one host but not in the other [47]. As mentioned, high interplant variation could be one of the causes, but a more significant one, is the high gene flow that might prevent genetic differentiation among hosts [20], at least for adaptive loci. Although *B. campestris* is the best host for *B. brassicae*, the high local abundance of the cultivated *B. oleracea* is a constant source, albeit less suitable, for aphids to colonize thus retarding/limiting the evolution of host local adaptation.

The host plant species plants provide distinct selective environments for *B. brassicae* and the optimal phenotype is different in each. The average phenotypic response occurs through plasticity at low fitness cost. van Tienderen [38] showed that in a coarse-grained environment consisting of two types of habitats, a population under soft selection is expected to evolve towards a compromise between the reaction that would be optimal within each habitat and a cost-free reaction norm, then predicted field populations composed of specialists, generalist, or intermediates or in a transitory state. This scenario may be represented by populations of *B. brassicae* in the Chiapas highlands where 20 genotypes reached the highest intrinsic growth rate in *B. campestris*; six genotypes performed better in *B. oleracea*, and six were generalists or canalized genotypes (i.e., equal intrinsic growth rate on both hosts). The specialists can use both hosts at nil or low cost of phenotypic plasticity. The relationship between fitness and plasticity in size (PC1) and relative body length (PC2) was positive and marginally significant. Plastic genotypes increased their fitness ca. 9.3%. We found a high correlation between the mean trait value and trait plasticity (PC1, PC2), but distinct between host plants (cf. Auld [36]). Trait value and its plasticity were positively correlated in *B. oleracea* but negatively in *B. campestris*, suggesting a biased estimate of the possible costs of plasticity. In both plants, more plastic genotypes had the most extreme phenotype, although in the opposite direction [36]. Since larger general size renders higher fitness in *B. campestris*, plasticity covariate negatively with trait value and hence with fitness, resulting in a weak relationship between fitness and trait plasticity. The lack of a relationship between residual fitness and trait plasticity implies absence and/or nil cost of plasticity [16], [36], [38]. Thus it is possible that selection has already removed genotypes of *B. brassicae* that incurred high costs of plasticity [48] or that plasticity in *B. brassicae* represent “noisy plasticity” [49]. It appears that selection in each host is counterbalanced by plasticity.

We detected selection on aphid size (PC1) in *B. campestris* and *B. oleracea* indicating that the average phenotype is suboptimal in each host, while the relative body length is suboptimal in *B. oleracea* but not in *B. campestris* [38]. This result reinforces the idea that these host species represent distinct selective environments for *B. brassicae*, as has been reported in previous studies [20], [39]. Larger aphids attain higher fitness in both hosts but the average size is higher in *B. campestris* and thus the highest fitness is observed in this host, suggesting potential for host specialization of *B. brassicae* to *B. campestris*.

Host specialization of phytophagous insects is a relatively common phenomenon [50], [51], and may be a prelude of adaptive speciation [52]. Disruptive selection has been considered as the main selection mode driving speciation under sympatric conditions[53]–[56]. We failed to detect whether this mode of selection, due to its relationship with fitness, affects size. Directional selection between hosts may promote differentiation if the selection differs in intensity [57]. This proved to be true for *B. brassicae*, but perhaps selection intensity has not been strong enough to produce stronger genetic and phenotypic divergence [13], producing local adaptation to the host under sympatric condition [15]. An additional constraint for evolution of local adaptation of *B. brassicae* in the Chiapas highlands is reproduction by parthenogenesis. Under this reproductive system the emergence of reproductive barriers necessary for adaptive speciation is precluded. Furthermore, as mentioned previously, there is a constant gene flow, via colonization, between host-associated populations [24], such that when coupled with plasticity, reduces the genetic differentiation [58].

In this study, phenotypic plasticity seems a sufficient for preventing the erosion of genetic variation by natural selection [11], [12], [59], delaying or impeding herbivore’s host race formation. Higher genotype×environment in relation to host use and strong selection within hosts could promote host race formation and incipient sympatric speciation, as has been documented in other aphid species, *Acyrtosiphum pisum* [15], [52] *Aphis fabae* [60] and *Myzus persicae* [61], [62], although in the case of *B. brassicae* it seems to be a limited evolutionary path.

## Supporting Information

Table S1 **Original data set of morphologic and life history measure of** ***Brevicoryne brassicae*** **genotypes reared on** ***Brassica oleracea*** **and** ***Brassica campestris*** **.**
(XLSX)
